# Pathological findings associated with *Dipetalonema* spp. (Spirurida, Onchocercidae) infection in two species of Neotropical monkeys from Brazil

**DOI:** 10.1007/s00436-023-07895-3

**Published:** 2023-06-22

**Authors:** Luiza Presser Ehlers, Mônica Slaviero, Cíntia De Lorenzo, Renata Fagundes-Moreira, Viviane Kelin de Souza, Lívia Perles, Vinicius Baggio-Souza, Marcos Antonio Bezerra-Santos, David Modrý, Michal Benovics, Welden Panziera, David Driemeier, Saulo Petinatti Pavarini, João Fabio Soares, Domenico Otranto, Luciana Sonne

**Affiliations:** 1 Setor de Patologia Veterinária, Faculty of Veterinary, Universidade Federal do Rio Grande do Sul (UFRGS), Porto Alegre, Rio Grande do Sul Brazil; 2 Laboratório de Protozoologia e Rickettsioses Vetoriais, Faculty of Veterinary, Universidade Federal do Rio Grande do Sul (UFRGS), Porto Alegre, Rio Grande do Sul Brazil; 3 Department of Veterinary Medicine, University of Bari, Valenzano, Italy; 4 Department of Botany and Zoology, Faculty of Science, Masaryk University, 61137 Brno, Czech Republic; 5 Department of Veterinary Sciences, Faculty of Agrobiology, Food and Natural Resources/CINeZ, Czech University of Life Sciences Prague, 16500 Praha-, Suchdol, Czech Republic; 6 Biology Center, Institute of Parasitology, Czech Academy of Sciences, 37005 Ceske Budejovice, Czech Republic; 7 Faculty of Veterinary Sciences, Bu-Ali Sina University, Hamedan, Iran

**Keywords:** Onchocercidae, Vector-borne nematodes, Filariasis, *Alouatta guariba clamitans*, *Sapajus nigritus*, Pathology

## Abstract

Among vector-borne helminths, filarioids of the genus *Dipetalonema* (Spirurida: Onchocercidae) localize in several tissues and body cavities of several animal species, causing mild to moderate lesions. The pathological findings associated with *Dipetalonema* spp. infection in Neotropical monkeys from southern Brazil are herein described, along with a fatal case due to filarial polyserositis and entrapment of an intestinal segment. At necropsy, nematodes were observed in abdominal and thoracic cavities, or in the pericardium of 37 (31.3%) out of the 118 individuals examined (i.e., 35 *Alouatta guariba clamitans* and two *Sapajus nigritus*). In addition, at histology, 27.0% of positive animals presented microfilarie (inside blood vessels of lung, spleen, liver, and brain) and 8.1% presented adult nematodes in the heart, lung, and liver. In two cases, cross-sections of filarioids were associated with areas of epicardial thickening with intense fibrosis and pyogranulomatous inflammation in the brain, heart, liver, lungs, or spleen. The DNA fragment was amplify using the *cox*1 gene, sequenced and analyzed to identify the nematode species collected; presence of *Wolbachia* was assessed in the filarioids using the 16S rRNA gene. At BLAST analysis of the *cox*1 gene, 10 sequences showed 91.7% nucleotide identity with *Dipetalonema gracile*, and two with *D. gracile* (98.5%) and *Dipetalonema graciliformis* (98.3%). Phylogenetic analyses clustered sequences of the *cox*1 obtained in this study in two clades corresponding with the host species. *Wolbachia* sp. endosymbiont was detected in four samples. Data herein reported provide a description of pathological lesions associated with the infection by *Dipetalonema* spp., suggesting that they may cause disease in Neotropical monkeys. In addition, a better understanding of diversity and biology of *Dipetalonema* spp. in South America is needed to assess the impact they may cause in native non-human primates from Brazil.

**Supplementary Information:**

The online version contains supplementary material available at 10.1007/s00436-023-07895-3.

## Introduction

Filarioids of the genus *Dipetalonema* (Spirurida: Onchocercidae) are parasites localized in the subcutaneous, body cavities, and other tissues of many animal species (Travi et al. 1985; Notarnicola et al. 2008), being vectored by arthropods, mainly of the genus *Culicoides* (Eberhard et al. 1979). These filarioids have been primarily described in wildlife animals, being most of them regarded as of minor or none pathogenicity (Travi et al. 1985; Karesh et al. 1998). In the Neotropical region, some *Dipetalonema* species (i.e., *D. gracile, D. graciliformis, D. freitasi*, *D. caudispina*, *D. yatesi*, and *D. robini*) have been collected in body cavities and several tissues (e.g., subcutaneous, lung, spleen) from monkeys of the genera *Ateles*, *Cebus*, *Sapajus*, *Saimiri*, *Lagothrix*, and *Saguinus* (Strait et al. 2012; Lefoulon et al. 2015; Vanderhoeven et al. 2017; Conga et al. 2018; Zárate-Rendón et al. 2022). Though primates infected with these filarioids do not display clinical signs, pathological lesions such as pleuritis, fibrinopurulent peritonitis, and fibrinous adhesion have been occasionally described (Travi et al. 1985; Strait et al. 2012; Baker 2019).

Neotropical monkeys are a diverse group of arboreal primates that inhabit tropical forests (Rosenberg and Hartwig 2013). In Brazil, monkeys of the genera *Alouatta* and *Sapajus* are distributed in different biomes, being often threatened due to anthropic pressures (e.g., dog attacks, hunting, road kills, and electric shock), as well as vector borne diseases, such as yellow fever (Moreno et al. 2015; Ehlers et al. 2022). Studies about the impact of filarioids on these animal species are scant and mainly limited to accidental macroscopic lesions (Bueno et al. 2017; Lopes et al. 2022). In this study, we describe pathological findings associated with *Dipetalonema* spp. infection in a large number of individuals sampled under the frame of a surveillance project on infectious diseases of Neotropical monkeys.

## Methods

### Sample collection

From April 2017 to October 2021, *Alouatta guariba clamitans* (*n* = 107) and *Sapajus nigritus* (*n* = 11) monkeys dead for different causes (e.g., dog attacks, road kills, electric shock, yellow fever, pneumonia, pleuropneumonia, hemorrhagic colitis, chronic renal failure, bacterial meningoencephalitis, and neoplasms) were collected and delivered to the Veterinary Pathology section of the Universidade Federal do Rio Grande do Sul for *post-mortem* examination. Animals came from zoological gardens, wildlife rescue centers, institutes of protection of wildlife, wildlife keepers, and veterinary clinics from Southern Brazil (see the “Acknowledgments” section).

### Pathological and molecular analysis

Adult nematodes were collected from abdominal cavity, thoracic cavity, and/or pericardium, and subsequently frozen for further analysis. Nematodes collected at the necropsy were stored for further examination and fragments of organs (i.e., brain, diaphragm, heart, liver, lung, and spleen) were collected and fixed in 10% formalin solution. Afterwards, samples were embedded in paraffin, cut in 3-μm-thick slices and stained using the hematoxylin and eosin (HE). Genomic DNA from 12 filarioids (*n=*10 from *A. guariba*; *n*= 2 from *S. nigritus*) was extracted using the commercial kit Pure Link® Genomic DNA Mini Kit (Invitrogen ^TM^, Carlsbad, CA, EUA) according to the manufacturer’s instructions. Samples were processed by conventional PCR (cPCR) assay using primers amplifying a portion (~689 bp) of partial mitochondrial cytochrome *c* oxidase subunit 1 (*cox*1) (Casiraghi et al. 2001). *Thelazia callipaeda* DNA was used as a positive control in PCR assays. In addition, samples were tested for *Wolbachia* spp. DNA through a cPCR targeting the 16S rRNA gene (Parola et al. 2003) and all PCR products were visualized by UV transilluminator following electrophoresis in 2% red-stained agarose gel.

Amplicons of the expected size were purified and sequenced in both directions using the Big Dye Terminator v.3.1 chemistry in a 3130 Genetic Analyzer (Applied Biosystems, California, USA) in an automated sequencer (ABI-PRISM 377). Consensus sequences were edited and compared with reference sequences available on GenBank database using the Basic Local Alignment Search Tool (BLAST). For each gene, sequences were selected for phylogenetic inferences based on BLAST results. The final dataset included 33 sequences, with *Thelazia callipaeda* selected as outgroup, and was aligned using the Fast Fourier transform algorithm in MAFFT (Katoh et al. 2019) using G-INS-I refinement method, and the ends were manually trimmed to unify their length. All parameters for phylogenetic analyses were treated as variables; therefore, GTR (the general time-reversible evolutionary model) was selected as the preferred evolutionary model to not *a priori* reduce the heuristic search. The shape parameter of the gamma distribution (G) and the proportion of invariable sites (I) was selected using jModelTest v 2.1.10 (Guindon and Gascuel 2003, Darriba et al. 2012), and the data were treated as partitioned, computing and applying the optimal substitution model for each position within codon individually. Phylogenetic analyses using maximum likelihood (ML) were computed employing RAxML v 8.1.12 (Stamatakis 2006, 2014). The best ML tree was selected from 100 iterations, and support for the branching pattern was validated through 10^3^ pseudoreplicates. Phylogenetic analyses of Bayesian inference (BI) were carried out in MrBayes v 3.2 (Ronquist et al. 2012), and the resulting tree was constructed using the Metropolis-coupled Markov chain Monte Carlo algorithm. Four concurrent chains (one cold and three heated) ran for 10^6^ generations, sampling trees every 100 generations. The first 30% of trees were discarded as a relative burn-in period after checking that the standard deviation split frequency fell below 0.01. Results were checked in Tracer v 1.7.1 (Rambaut et al. 2018) to assess convergence. Posterior probabilities were calculated as the frequency of samples recovering particular clades. The best evolutionary model was chosen under the Akaike information criterion using the iqTREE software (available at: http://iqtree.cibiv.univie.ac.at/). The phylogenetic analysis was performed by Bayesian analysis using CIPRES gateway (Ronquist and Huelsenbeck 2003). Protein translation for *cox*1 gene was performed with MEGA 11 software (Kumar et al. 2018) to check for whether the sequences contain incorrectly recognized nucleotides. Interspecific and intraspecific nucleotide divergence and the presence of haplotypes were evaluated according to criteria previously established by Ferri et al. (2009).

## Results

**Fig. 1:**
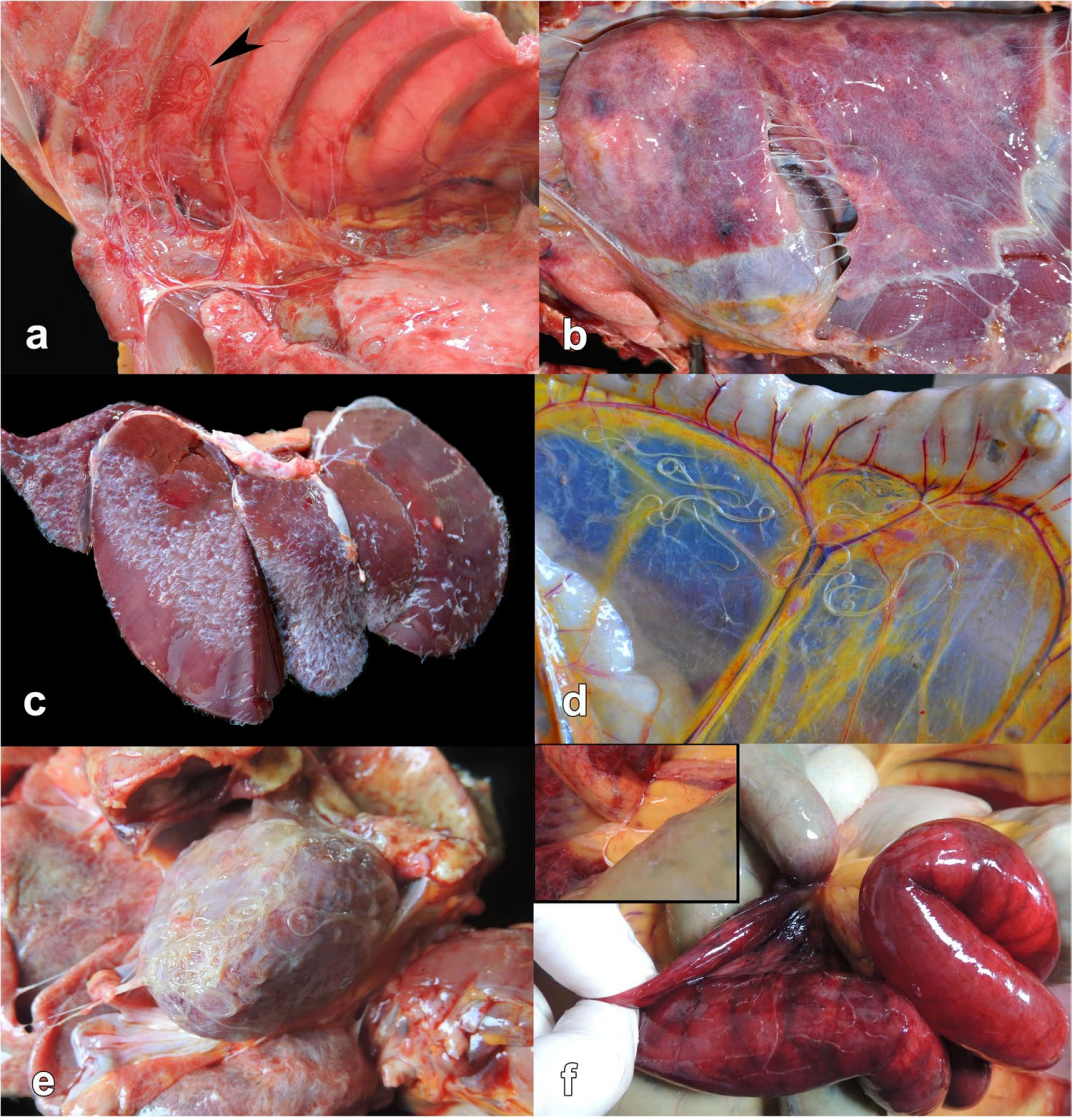
Gross lesions in *Alouatta guariba clamitans* and *Sapajus nigritus *monkeys infected by *Dipetalonema* spp. (**a**; case 2) Thoracic cavity with multifocal areas of fibrous adhesions in the visceral and parietal pleura associated with filarial nematodes (arrowhead) in an individual with polyserositis. (**b**; case 12) Thoracic cavity with proliferation of fibrous connective tissue in the visceral pleura causing adhesions in the lung. (**c**; case 7) Liver, marked proliferation of fibrous connective tissue in the form of fringes over the organ capsule. (**d**; case 13) Abdominal cavity, filarial nematodes in the mesentery. (**e**; case 13) Heart with epicardium presenting pale multifocal areas, and moderate adherence by fibrous and fibrinous serositis associated with filarial nematodes. (**f**; case 20) Small intestine with entrapment of intestinal segment by focal area of fibrosis with fibrous polyserositis caused by filarial nematodes

Filarioid nematodes were detected in 37 (31.3%) individuals out of 118 primates necropsied, of which 35 were Southern Brown Howler Monkey (*A. clamitans*) and two Black-horned Capuchin (*S. nigritus*). Pathological gross lesions included polyserositis which it was characterized by proliferation of fibrosis (*n* = 31/37; 83.8%), and deposition of fibrinous material (*n* = 18/37; 48.6%) (Fig. 1a, b, c). In addition, serosa adhesion (*n* = 19/37; 51.4%) and hydrothorax (*n* = 9/37; 24.3%) were observed. Filiform nematodes (3.0–10.0 cm in length) were associated with polyserositis and were free and/or adhered to the surface of organs, in the abdominal cavity (Fig. 1d), thoracic cavity, and pericardium (Fig. 1e). In the intestine (*n* = 1/37; 2.7%), a focal area of fibrosis causes entrapment of the distal portion of the jejunum and the initial portion of the ileum (Fig. 1f). Characteristics and frequency of the lesions observed in the animals are reported in Table 1 and Supplementary file 1.

**Table 1:** Main pathological findings (gross and histopathological) described in different anatomical locations of Neotropical primates along with their frequency

Localization	Lesion	Frequency (*n*; %) (*n*=37)
Abdominal	Polyserositis (G)	31; 83.8%
	Adult nematodes (G)	34; 91.9%
Thoracic	Polyserositis (G)	21; 56.8%
	Adult nematodes (G)	14; 37.8%
Pericardium	Polyserositis (G)	8; 21.6%
	Adult nematodes (G)	5; 13.5%
Epicardium	Multifocal pale areas (G)	8; 21.6%
	Fibrosis (HE)	7; 18.9%
	Fibrin deposition (HE)	3; 8.1%
	Cross sections of adult nematodes (HE)	2; 5.4%
Myocardium	Multifocal pale areas (G)	1; 2.7%
	Fibrosis (HE)	1; 2,7%
Lungs	Pleural thickening (G)	19; 51.3%
	Reddish multifocal areas (G)	9; 24.3%
	Deposition of fibrinous material (G)	4; 10.8%
	Fibrosis (HE)	18; 48.6%
	Fibrin deposition (HE)	7; 18.9%
	Alveolar septa thickened (HE)	6; 16.2%
	Microfilariae (HE)	6; 16.2%
	Cross sections of adult nematodes (HE)	1; 2.7%
Liver	Enlargement and pallor (G)	5; 13.5%
	Thickened Glisson's capsule (G)	5; 13.5%
	Fibrosis (HE)	5; 13.5%
	Microfilariae (HE)	4; 10.8%
	Fibrin deposition (HE)	2; 5.4%
	Cross sections of adult nematodes (HE)	1; 2.7%
Mesentery	Focal area of fibrosis, causing ischemic necrosis of intestine segment (G)	1; 2.7%
Spleen	Microfilariae (HE)	4; 10.8%
	Thickening of the capsule (G)	3; 8.1%
	Fibrosis (HE)	3; 8,1%
	Fibrin deposition (HE)	2; 5.4%
Brain	Microfilariae (HE)	1; 2.7%

*G* gross findings, *HE* histopathological findings

**Fig. 2:**
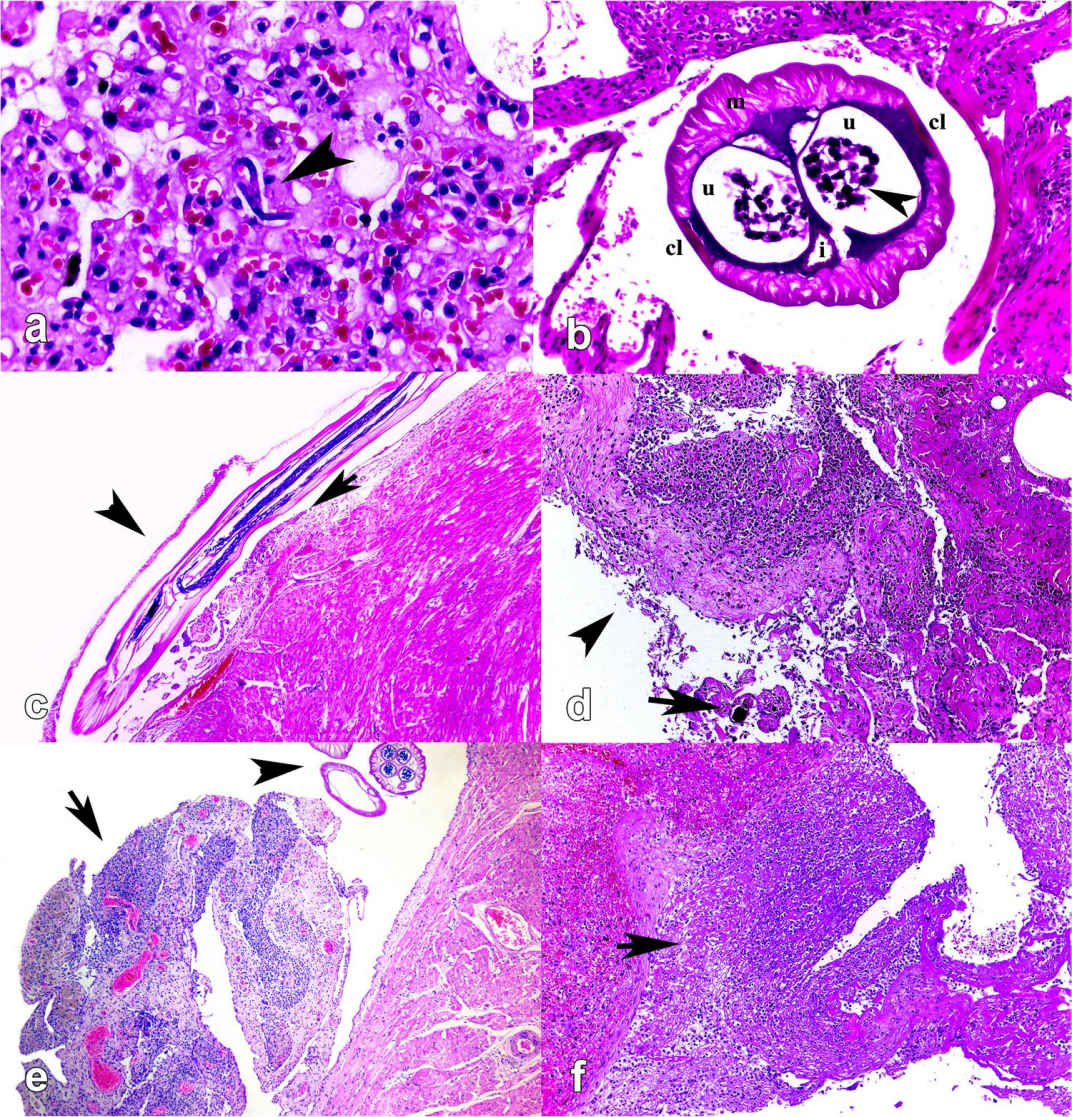
Histological findings of *Dipetalonema* spp. infection in *Alouatta guariba clamitans* and *Sapajus nigritus*. (**a**; case 13) Lung, longitudinal section of 15 to 30 μm microfilariae in the lumen of capillary in alveolar septum. Hematoxylin and eosin (HE), × 400. (**b**; case 24) Cross section of adult female filarial nematode showing coelomyarian muscles (m), lateral cords (cl), intestine (i), and uterus (u) with developing microfilariae (arrowhead), attached to fibrous and fibrinous serositis associated with a moderate pyogranulomatous infiltrate. HE, × 400. (**c**; case 32) Heart, longitudinal section of an adult filarial nematode adhered to the epicardium (arrowhead) and associated with mild fibrous serositis (arrow). HE, × 40. (**d**; case 13) Lung, moderate thickening of visceral pleura due to proliferation of fibrous connective tissue and fibrin deposition, lymphohistiocytic and eosinophilic infiltrate (arrowhead) and focal mineralization (arrow). HE, × 200 (**e**; case 13) Heart, fibrous, and fibrinous serositis associated with inflammation (arrow), and filarial nematode cross-sections (arrowhead). HE, × 40. (**f**; case 32) Spleen, fibrous and fibrinous serositis associated with an intense pyogranulomatous infiltrate (arrow). HE, ×100

At the histology, microfilariae (*n* = 10/37; 27.0%) were detected inside blood vessels and capillaries of multiple organs, including the lung (Fig. 2a), spleen, liver, and brain, filled with multiple basophilic structures. Furthermore, cross-sections of adult nematodes (*n* = 3/37; 8.1%) covered by eosinophilic cuticle, with apparent coelomyarian muscles and presenting lateral cords, were observed (Fig. 2b), digestive and reproductive tracts. Nematodes were associated with areas of polyserositis in the heart (Fig. 2c), lung, and liver. In the lungs, pleural lesions were observed (Fig. 2d), being thickened due to fibrosis, fibrin deposition, and inflammatory pattern of different nature (i.e., lymphohistiocytic and eosinophilic, pyogranulomatous or neutrophilic). Epicardial thickening was characterized by intense fibrosis (Fig. 2e), fibrin deposition, associated with reactive mesothelial cells and predominantly lymphohistiocytic and eosinophilic inflammatory infiltrate. In two cases, cross-sections of adult forms of filarioids were observed in the epicardium.

Liver presented thickened Glisson’s capsule, characterized by fibrosis covered by hyperplastic mesothelial cells, with fibrin deposition associated with a lymphohistiocytic and eosinophilic or pyogranulomatous inflammatory infiltrate. Similarly, longitudinal sections of microfilariae in the periportal region were associated with a mild inflammatory infiltrate, being predominantly lymphohistiocytic and eosinophilic.

Thickening of the spleen capsule was characterized by fibrosis, hyperplastic mesothelial cells, and fibrin deposition associated with a slight lymphohistiocytic and eosinophilic or pyogranulomatous inflammatory infiltrate (Fig. 2f).

At the BLAST analysis of the *cox*1 gene, nucleotide identity ranged from 91.7% with *D. gracile* (Accession number: KP760179) in 10 entries (i.e., detected in *A. clamitans*), to 98.5% and 98.3% with *D. gracile* (Accession number: KP760180) and *D. graciliformis* (Accession number: KP760182), in two sequences (i.e., detected in *S. nigritus*), respectively (Table 2). The *cox*1 sequences from the filarioids collected in the two primate species differed by the presence of seven silent mutations present in the two sequences from the specimens collected in *S. nigritus*. Based on this, the molecular results confirmed the species identification as *Dipetalonema* spp. *Wolbachia* sp. endosymbiont was detected in four samples (*n*= 2 *A. guariba*; *n*= 2 *S. nigritus*) presenting 99.4% nucleotide identity (100% query coverage) with *Wolbachia* sp. endosymbiont from *D. gracile* (Accession number: KU255234).

**Table 2:** Species identification and GenBank accession numbers for *cox*1 gene sequences obtained from filarioids of *Alouatta guariba clamitans* and *Sapajus nigritus*

Monkey species	Sample ID	Fragment size (bp)	Pathogen similarity	% identity	GenBank accession number**
*A. clamitans*	Lami3A	482	*Dipetalonema gracile*	91.7% (KP760179)	OQ508916
*A. clamitans*	Lami3T	482	*Dipetalonema gracile*	91.7% (KP760179)	OQ508908
*A. clamitans*	Cascata5A	482	*Dipetalonema gracile*	91.7% (KP760179)	OQ508912
*A. clamitans*	Viamao12A	482	*Dipetalonema gracile*	91.7% (KP760179)	OQ508911
*A. clamitans*	StAntonio Patrulha13S	482	*Dipetalonema gracile*	91.7% (KP760179)	OQ508917
*A. clamitans*	Gravatai14S	482	*Dipetalonema gracile*	91.7% (KP760179)	OQ508915
*A. clamitans*	Itapua16T	482	*Dipetalonema gracile*	91.7% (KP760179)	OQ508913
*A. clamitans*	Lami24S	482	*Dipetalonema gracile*	91.7% (KP760179)	OQ508910
*A. clamitans*	Canoas29T	482	*Dipetalonema gracile*	91.7% (KP760179)	OQ508909
*S. nigritus*	SapucaiaSul31A	482	*Dipetalonema gracile* *Dipetalonema graciliformis*	98.5% (KP760180) 98.3% (KP760182)	OQ508918
*A. clamitans*	Barrado Ribeiro34A	482	*Dipetalonema gracile*	91.7% (KP760179)	OQ508914
*S. nigritus*	RS37A	482	*Dipetalonema gracile* *Dipetalonema graciliformis*	98.5% (KP760180) 98.3% (KP760182)	OQ508919

*NA not amplified

**Sequence accession numbers of the present study deposited in GenBank

**Fig. 3:**
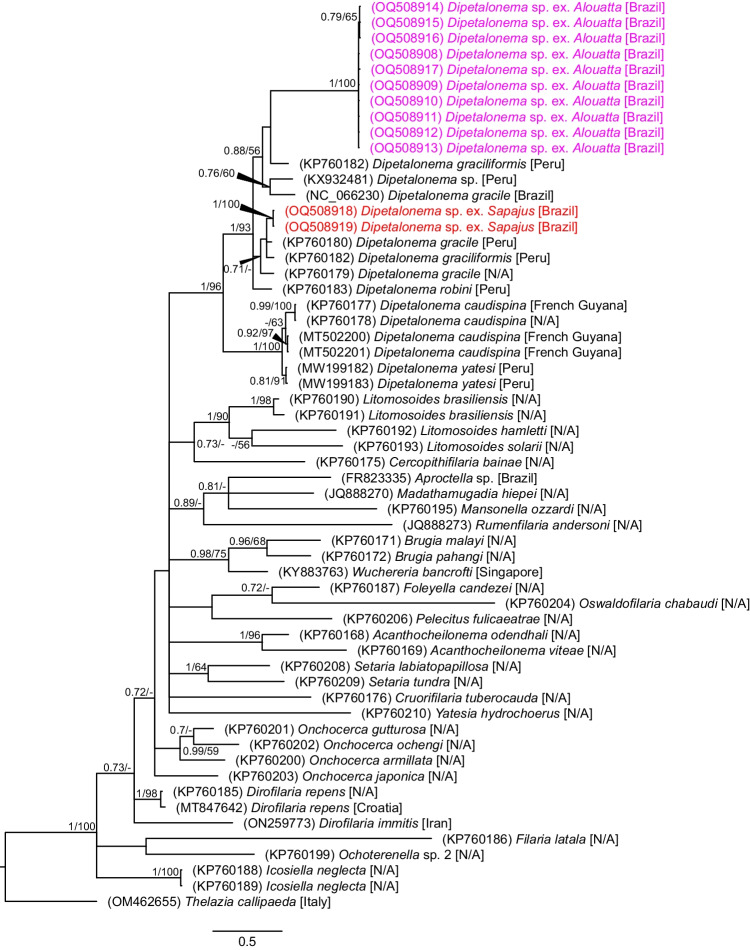
Phylogenetic tree of filarioid species built from partial sequences of *cox*1 gene. Values at the nodes indicate posterior probabilities from BI and bootstrap values from ML analyses. Dashes indicate values below 0.70 and 50, respectively. Colored are sequences obtained from *Dipetalonema* sp. specimens within this study: violet = specimens from *Alouatta guariba clamitans*; red = specimens from *Sapajus nigritus*

The final alignment for phylogenetic analyses spanned 610 unambiguously aligned nucleotide positions and the results of both statistical analyses (BI and ML) showed that nucleotide sequences obtained from this study clustered in two clades, ten in a single separate clade, and two in a clade with *D. graciliformis* (Fig. 3). All sequences obtained in this study were submitted in the GenBank database under the accession numbers: OQ508908 to OQ508919 (*cox*1), and OQ536153 to OQ536156 (16S rRNA).

## Discussion

Data presented indicate that *Dipetalonema* spp. infections in Neotropical monkeys from Southern Brazil are associated with pathological lesions, such as those in the pericardium. In addition, a case of entrapment of an intestinal segment due to massive infection by these filarioids was also described, resulting in the death of a monkey (i.e., *A. clamitans*). Filarioids were mostly found in the abdominal cavity, as also reported in previous studies of *Dipetalonema* spp. in Neotropical primates (Notarnicola et al. 2008; Corrêa et al. 2016; Vanderhoeven et al. 2017; Lopes et al. 2022). *Dipetalonema* spp. have been morphologically described in *A. guariba* and *Sapajus flavius* from Rio Grande do Sul and Paraíba states, respectively (Bueno et al. 2017; Lopes et al. 2022), and *D*. *gracile* was molecularly characterized in *Saguinus bicolor* from Amazonas (Costa et al. 2023). Nonetheless, none of the above studies reported pathological findings. The presence of filarioids inside the pericardium seems to be the cause of major lesions in the epicardium, due to the intense fibrin deposition and fibrosis in eight primates. In addition, focal area of fibrosis in the mesentery of one individual may have been caused by the migration of the parasites, resulting in ischemic necrosis of the intestinal portion with consequent death of the monkey.

Pathological lesions associated with the presence of similar filarioid species have been occasionally described in humans. For example, chronic fibrous and fibrinous pericarditis were reported in people infected with *Mansonella perstans* adult worms (Simonsen et al. 2011; Mediannikov and Ranque 2018). In addition, polyserositis observed in animals from this study are similar to those described for *M. perstans* in humans, and by *Litomosoides sigmodontis* in rodents (Fercoq et al. 2019; Fercoq et al. 2020). Indeed, lesions caused by both nematode species have been associated with the development of the adult filarioids in the cavities (Jaquet 1980; Fercoq et al. 2020).

Infections by *Dipetalonema* spp. have been previously described in Neotropical primates causing mild peritonitis or chronic pleuritis, which are associated with areas of fibrous and occasionally, fibrinous adhesions (Chalifoux 1993; Strait et al. 2012). Similarly, other pathological findings (e.g., polyserositis, peritonitis, eosinophilic or lymphocytic infiltration) have been associated with filarial nematodes (i.e., *Setaria tundra*, *Onchocerca flexuosa*) in other hosts such as cervids from Finland, Japan, and Poland (Laaksonen et al. 2009; Kowal et al. 2013; Abd-Ellatieff et al. 2022). However, further description of the lesions was not performed to confirm whether the injuries were caused by the presence of the nematodes in the abdominal cavity (Laaksonen et al. 2007; Nikander et al. 2007).

The pathological lesions in the animals herein described could be an important health issue associated with *Dipetalonema* spp., since the filarioids infecting the primates from this study may cause clinical disease or even death depending on the infection intensity. These primates native to South America are considered threatened species (MMA 2014; Jerusalinsky et al. 2020), with populations affected by anthropic activities (e.g., dog attacks, hunting, road kills, and electric shock), and endemic diseases such as yellow fever (Chiarello and Galetti 1994; Bicca-Marques and de Freitas 2010). Therefore, confirming whether ubiquitous filarioids may represent a health problem for monkeys is of importance as a primary or concomitant cause of diseases in infected animals. In addition, though *Wolbachia* endosymbionts are implicated in inflammatory-mediated filarial infections (Taylor 2003; Manoj et al., 2021), its finding did not allow to draw any conclusions about their role in the occurrence of the lesions herein described.

Obtained sequences of the *cox1* clearly separated the filarioids from *A. clamitans* and *S. nigritus* into separate clades. This fact, together with the low nucleotide identity with *D. gracile* (i.e., 91.7%), suggests that the specimens from *A. clamitans* may belong to a separate, presumably new, taxon within the genus *Dipetalonema*. Further studies should address in detail the morphology of the adult nematodes combined with further molecular analyses to confirm their taxonomic status and described this filarioid as a new species within the genus *Dipetalonema.*

## Conclusion

Data herein reported provide detailed description of pathological lesions associated with the infection by filarioids of the genus *Dipetalonema*, proving that these nematodes are able to cause disease in free ranging Neotropical monkeys. Understanding the life cycle, vectors, and transmission ecology of these nematodes in local ecological context, together with more material from free-ranging primates, can answer arising questions about possible impact of filarioid parasites on populations of free-ranging primates, especially in a case of fragmented populations of threatened species.

## Supplementary information

ESM 1

## Data Availability

The authors declare that data supporting the findings of this study are available within the article.
